# A Population Accounting Approach to Assess Tourism Contributions to Conservation of IUCN-Redlisted Mammal Species

**DOI:** 10.1371/journal.pone.0044134

**Published:** 2012-09-12

**Authors:** Ralf C. Buckley, J. Guy Castley, Fernanda de Vasconcellos Pegas, Alexa C. Mossaz, Rochelle Steven

**Affiliations:** International Centre for Ecotourism Research, Griffith University, Gold Coast, Queensland, Australia; University of Kent, United Kingdom

## Abstract

Over 1,000 mammal species are red-listed by IUCN, as Critically Endangered, Endangered or Vulnerable. Conservation of many threatened mammal species, even inside protected areas, depends on costly active day-to-day defence against poaching, bushmeat hunting, invasive species and habitat encroachment. Many parks agencies worldwide now rely heavily on tourism for routine operational funding: >50% in some cases. This puts rare mammals at a new risk, from downturns in tourism driven by external socioeconomic factors. Using the survival of individual animals as a metric or currency of successful conservation, we calculate here what proportions of remaining populations of IUCN-redlisted mammal species are currently supported by funds from tourism. This proportion is ≥5% for over half of the species where relevant data exist, ≥15% for one fifth, and up to 66% in a few cases. Many of these species, especially the most endangered, survive only in one single remaining subpopulation. These proportions are not correlated either with global population sizes or recognition as wildlife tourism icons. Most of the more heavily tourism-dependent species, however, are medium sized (>7.5 kg) or larger. Historically, biological concern over the growth of tourism in protected areas has centered on direct disturbance to wildlife. These results show that conservation of threatened mammal species has become reliant on revenue from tourism to a previously unsuspected degree. On the one hand, this provides new opportunities for conservation funding; but on the other, dependence on such an uncertain source of funding is a new, large and growing threat to red-listed species.

## Introduction

Threatened species survive largely in parks; parks need money to remain operational; and some of that money comes from tourism. Therefore, tourism contributes to the conservation of these species in parks. We calculate here what proportions of remaining global populations of IUCN-redlisted mammal species effectively depend on tourism revenue. That is, we use the number of individual living animals, ie the sizes of remaining wild populations, as a basic metric or currency of *in-situ* conservation success; and we use the proportions of parks agency budgets derived from tourism revenue as a measure of tourism contributions. We find that tourism now contributes significantly to the survival of many red-listed mammal species. This reliance on tourism, however, now places their survival at risk from externally generated downturns in tourism.

Arresting the continuing global decline in biodiversity is a major and broadly agreed international goal [1]–[2]. Despite this, species extinctions continue [3]–[8]. Most threatened species survive mainly in public protected areas [8]–[12], but populations still decline [3], [8], [13]. Contributing factors include poaching, disease, disturbance, habitat clearance and encroachment, interactions with invasive species, and modified fire regimes. Many parks agencies, especially in biodiverse developing nations, lack adequate funds to combat these threats [14]–[16].

Especially over the past decade, parks budgets in a number of countries have come to rely increasingly on revenues associated with tourism; principally fees and prices charged to visitors by parks agencies for entry, activities, accommodation and purchases [17]–[19]. This applies particularly in developing nations with heavy dependence on international tourism. Tourism, however, is sensitive to socioeconomic factors such as wealth and safety. When it suffers externally generated downturns, parks agencies are suddenly without funds for operational conservation management. This creates new risks for rare species. The reality of such risk has been demonstrated in countries such as Madagascar, Nepal, Zimbabwe and elsewhere, where tourism collapsed following military coups, and many threatened species suffered greatly increased hunting and poaching in consequence [20]–[25].

Here we quantify these risks by calculating the numbers and hence the proportions of remaining individuals that rely on tourism revenue for conservation in parks. We acknowledge that the political and financial dynamics of individual protected areas, as well as the population dynamics and conservation measures for individual species, are often highly complex. Sources of parks funding, however, are largely substitutable: parks agencies incur both conservation and recreation management expenditures irrespective of income, and funds are reallocated internally. At global scale, therefore, the simple accounting approach adopted here provides a valid mechanism to measure the reliance of red-listed mammal species on tourism revenue.

**Figure 1:**
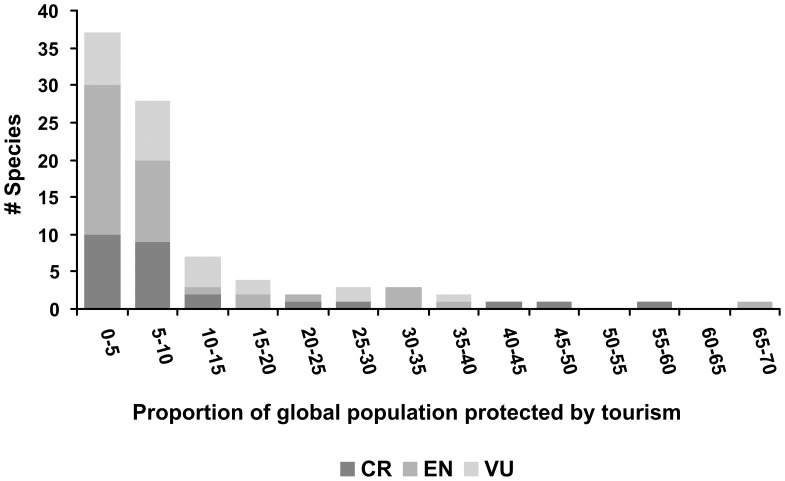
Proportions of remaining global populations of threatened mammal species for which conservation funding is derived from tourism revenues. CR, Critically Endangered; EN, Endangered; VU, Vulnerable.

**Table 1:** Proportions of globally threatened mammal species conserved through tourism revenues to protected areas.

Species	IUCN	Global population (G)	Number of protected populations	Numbers protected by tourism (SR)*	Proportion protected (T)
*Cercocebus galeritus*	EN	∼1200	2	793	66.1
*Cephalophus adersi*	CR	<1000	4	572	57.2
*Bubalus mindorensis*	CR	<250	1	117	46.7
*Rungwecebus kipunji*	CR	∼1000	3	409	40.9
*Cercocebus sanjei*	EN	<1300	2	476	36.7
*Rhynchocyon udzungwensis*	VU	15000–24000	2	8797	36.7
*Hippocamelus bisulcus*	EN	1500	?	512	34.1
*Axis calamianensis*	EN	<1000	1	318	31.8
*Nyctimene rabori*	EN	<2500	1	795	31.8
*Panthera leo*	VU	∼25000	>140	7227	28.9
*Loxodonta africana*	VU	∼500000	∼110	141371	28.3
*Beatragus hunteri*	CR	∼600	3	169	28.1
*Diceros bicornis*	CR	4880	30	1067	21.9
*Equus grevyi*	EN	1966–2447	7	490	20.0
*Equus zebra zebra*	VU	∼3000	17	554	18.5
*Rucervus duvaucelii*	VU	3500–5100	8	897	17.6
*Leontopithecus rosalia*	EN	1000	4	164	16.4
*Tapirus bairdii*	EN	<5500	14	895	16.3
*Hippopotamus amphibius*	VU	125000–148001	148	21015	14.2
*Setonix brachyurus*	VU	<10000	9	1804	14.1
*Bradypus pygmaeus*	CR	<5000	1	655	13.1
*Lycaon pictus*	EN	3000–5500	53	685	12.5
*Rhinoceros unicornis*	VU	2575	10	320	12.4
*Pseudalopex fulvipes*	CR	<250	2	28	11.4
*Pseudomys novaehollandiae*	VU	<10000	12	1134	11.3
*Macroderma gigas*	VU	7000–9000	1	860	9.6
*Burramys parvus*	CR	2250	2	213	9.5
*Lasiorhinus krefftii*	CR	115	1	11	9.4
*Potorous gilbertii*	CR	40	1	4	9.4
*Procolobus kirkii*	EN	<2000	1	183	9.2
*Lagostrophus fasciatus*	EN	<10000		851	8.5
*Pseudomys oralis*	VU	∼10000	9	850	8.5
*Lagorchestes hirsutus ssp*	VU	∼6000	3	484	8.1
*Sminthopsis aitkeni*	CR	<500	1	40	8.0
*Isoodon auratus barrowensis*	VU	>25000	2	1984	7.9
*Ursus maritmus*	VU	20000–25000	25	1917	7.7
*Pseudomys fieldi*	VU	2000	2	142	7.1
*Myotis sodalis*	EN	∼400000	9	27589	6.9
*Leontopithecus chrysopygus*	EN	1000	1	64	6.4
*Elephas maximus*	EN	41410–52345	>33	3294	6.3
*Mustela nigripes*	EN	500–1000	9	62	6.2
*Dipodomys insularis*	CR	∼100	1	6	5.9
*Procyon pygmaeus*	CR	<1000	?	59	5.9
*Acinonyx jubatus*	VU	7000–10000	19	566	5.7
*Phascogale pirata*	VU	<10000	3	567	5.7
*Onychogalea fraenata*	EN	450	3	24	5.2
*Bettongia penicillata*	CR	<7000	8	366	5.2
*Myrmecobius fasciatus*	EN	<1000	6	52	5.2
*Mesocapromys angelcabrerai*	EN	<2500	1	125	5.0
*Mesocapromys auritus*	EN	<2500	1	125	5.0
*Mysateles meridionalis*	CR	<250	1	12	5.0
*Natalus primus*	CR	∼100	0	5	5.0
*Perameles bougainville*	EN	<10000	1	497	5.0
*Porcula salvania*	CR	<500	2	24	4.8
*Propithecus perrieri*	CR	<250	1	11	4.6
*Brachyteles hypoxanthus*	CR	855	4	34	4.0
*Hapalemur aureus*	EN	∼1500	2	59	4.0
*Panthera tigris*	EN	3000–5000	>40	200	3.9
*Varecia variegata*	CR	<10000	9	391	3.9
*Leontopithecus caissara*	CR	400	2	14	3.6
*Panthera uncia*	EN	4080–6590	27	234	3.6
*Macaca silenus*	EN	<4000	17	136	3.4
*Melursus ursinus*	VU	<20000	>175	672	3.4
*Equus zebra hartmannae*	VU	25000	8	824	3.3
*Saguinus oedipus*	CR	∼6000	3	187	3.1
*Leporillus conditor*	VU	4000	4	124	3.1
*Nilgiritragus hylocrius*	EN	2000–2500	4	73	2.9
*Pseudomys fumeus*	EN	<2501	3	71	2.8
*Ammospermophilus nelsoni*	EN	124000–413000	4	11179	2.7
*Sarcophilus harrisii*	EN	10000–25000	?	661	2.6
*Leontopithecus chrysomelas*	EN	6001–15000	2	383	2.6
*Parantechinus apicalis*	EN	500–1000	6	26	2.6
*Procolobus gordonorum*	EN	10000–15400	1	367	2.4
*Cynomys parvidens*	EN	8000	1	189	2.4
*Propithecus tattersalli*	EN	6000–10000	1	229	2.3
*Cephalophus spadix*	EN	<1500	8	33	2.2
*Ailurus fulgens*	VU	∼10000	69	174	1.7
*Canis rufus*	CR	<150	3	2	1.6
*Crypytoprocta ferox*	VU	<2500	2	34	1.4
*Equus hemionus*	EN	∼24000	4	320	1.3
*Macrotis lagotis*	VU	<10000	6	131	1.3
*Blastocerus dichotomus*	VU	∼45000	2	587	1.3
*Romerolagus diazi*	EN	2478–12120	2	146	1.2
*Galidictis grandidieri*	EN	2650–3540	1	41	1.2
*Prolemur simus*	CR	<100	2	1	1.0
*Gymnobelideus leadbeateri*	EN	2000	1	19	0.9
*Urocyon littoralis*	CR	<1500	2	11	0.7
*Eulemur cinereiceps*	EN	∼7265	1	37	0.5
*Tapirus indicus*	EN	<5000		25	0.5
*Propithecus candidus*	CR	<250	2	1	0.2
					

* R: Argentina 26.5%, Australia 9.4%, Bolivia 8.1%, Botswana 81.1%, Brazil 7.8%, Canada 13.7%, Chile 37.9%, Colombia 7.6%, Costa Rica 18.2%, Cuba 5.0%, Guatemala 30.8%, Honduras 25.0%, India 8.0%, Kenya 66.1%, Madagascar 5.0%, Mexico 5.9%, Namibia 8.9%, Nepal 35.6%, Nicaragua 8.3%, Panama 13.1%, Philippines 53.0%, South Africa 47.2%, Tanzania 36.7%, Thailand 24.6%, United States 7.4%, Zambia 48.3%. Data from national parks agencies and international compendia (8,9).

**Figure 2:**
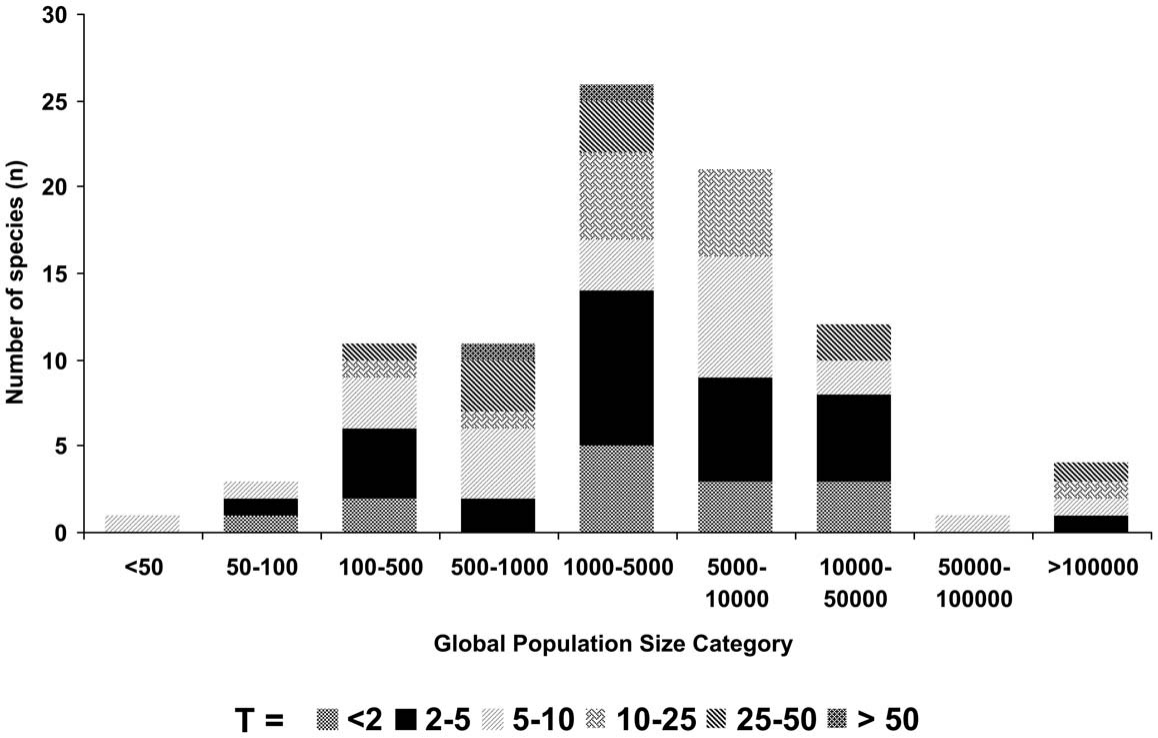
Global population sizes (maximum estimates) of threatened species, and proportions protected by tourism, T.

## Methods

For each species *a*, we calculated the proportions T of remaining individuals that rely on tourism revenue for conservation in parks, as T_a_ = Σ_i_^n^ S_ia_R_ia_/G_a_, where S_ia_ are subpopulation sizes and R_ia_ are proportions of revenue from tourism for the *i*th of the *n* parks in which species *a* occurs, and G_a_ is its global population. Higher T indicates greater dependence on tourism, and hence greater revenue-related risks to the threatened species concerned. R_ia_ are from gross revenues at national scale, since as noted above, agencies incur costs irrespective of income, and reallocate funds internally. In some nations, there are multiple categories of protected areas with different budget allocations per unit area; and in some agencies, budget data are available for individual parks. To maximise the number of species subpopulations for which both S and R data are available, however, we use the broadest and most widely available measure for R.

**Figure 3:**
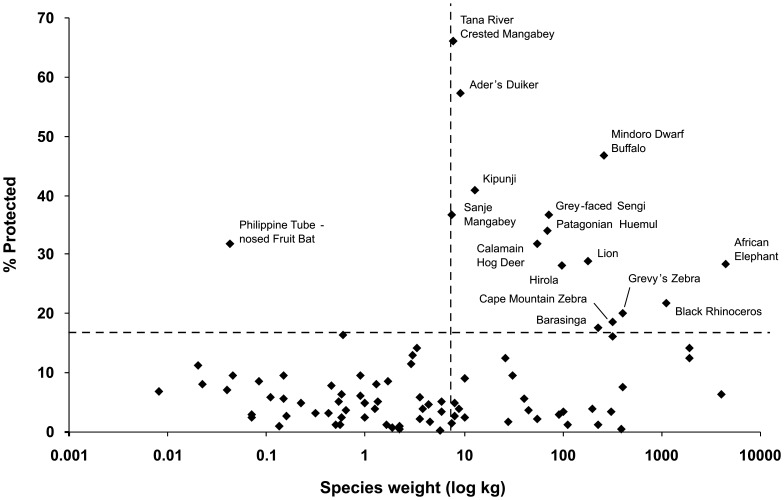
Proportions of populations dependent on tourism (T), relative to body weight. Dotted lines indicate 7.5 kg mean body weight, and 17% protected.

Subpopulation data S (Table S1) are derived from: IUCN Red Lists and supplementary materials; previous reviews [26]; and individual species conservation or recovery plans where available. Parks funding data R (Table S2) are from agency websites and the most recent available financial reports, audits and compendia. Data for R are more limited than for S. Data for R, S and G are available for 90 of the 1131 mammal species currently considered [26] as Vulnerable (VU), Endangered (EN), or Critically Endangered (CR). Data for G and S, but not R, are available for a further 52 species.

**Figure 4:**
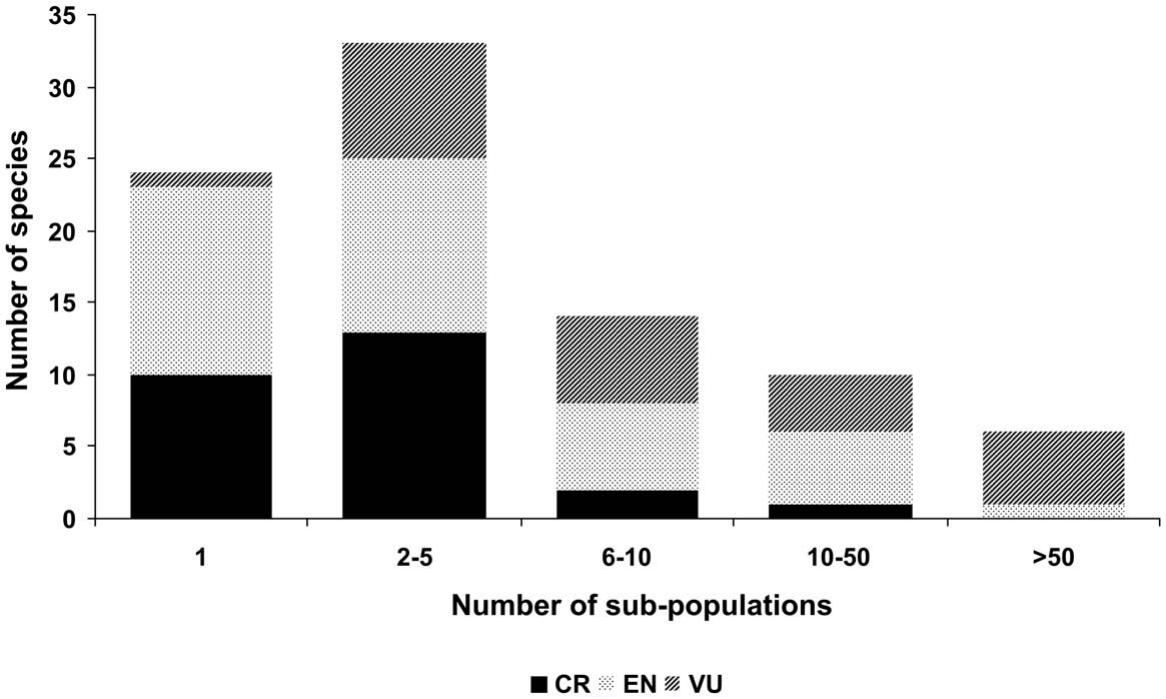
Numbers of extant subpopulations for species with known T.

For many species, data are only available for a subset of known subpopulations. Both the proportions of populations represented, and the reliability of the data concerned, differ considerably between species. For some individual species, reported population data may also change quite rapidly. IUCN Red Lists show common hippopotamus *Hippopotamus amphibius*, for example, as occurring in 138 protected parks, game, and nature reserves and sanctuaries. Subpopulation estimates, however, are available for only 20 of these, and financial data for only four. Ader’s duiker *Cephalophus adersi* occurs only in fragmented areas in Zanzibar and Kenya where local-scale population estimates are unreliable, so calculations are necessarily at national scale. For a few species such as black rhinoceros *Diceros bicornis*, subpopulation data are no longer released publicly because of poaching risks, and the data used here are compiled from country-level statistics.

The scarcity of data reflects the general paucity of information on threatened-species populations and parks-agency operations worldwide. The data presented here, however, are all that are currently available, and are more than adequate to demonstrate general patterns related to tourism revenues. Previous studies [6], [27] have faced similar deficiencies in data, but have yielded valuable assessments nonetheless. In particular, even though data are available for <10% of the IUCN-redlisted mammal species, there is no indication of any bias towards either more or less tourism-dependent species, as outlined below.

## Results

Of the 1131 IUCN-redlisted mammal species worldwide [25], data to calculate T are available for 90 (Fig. 1, Table 1). These data are derived from 379 subpopulations in 27 countries (Tables S1 and S2). Global population estimates for these species range from <50–500,000, with median ∼3300. T is not correlated with global population size (Fig. 2).

T≥5% for 58% of species with available data, T≥10% for 28%, and T≥15% for 20% (Fig. 1, Table 1). For two species, Ader’s duiker *Cephalophus adersi* and the Tana River crested mangabey *Cercocebus galeritus*, T>50%. That is, over half of the IUCN-redlisted mammal species listed in Table 1 rely on tourism to provide on-the-ground conservation funding for at least 5% of remaining individuals, and are hence at risk of at least a 5% population loss if tourism funding were to vanish as a result of a downturn in the industry. As noted earlier, in countries which have indeed experienced severe downturns in tourism, population losses of threatened species have indeed intensified: this is a very real mechanism. Population losses even at this scale are of global concern for any red-listed species. Rhino poaching in parts of Africa and Asia is currently causing annual losses around 2% of global populations, for example, and this is a topic of intense international public debate and global concern.

There is no correlation between reliance on tourism revenue, and recognition as a wildlife tourism icon, confirming that the data are not biased towards high-T species. Some tourism icon species, such as lion, one-horned rhinoceros and African elephant, have high T (≥10%), but others such as tiger, golden-headed lion tamarin, red panda and a number of lemur species, have low T (<5%) (Table 1). In addition, many high-T species such as the Patagonian huemul *Hippocamelus bisulcus* are not in themselves targets for wildlife tourists, but simply occur in scenically attractive parks. Most of the species with highest T (≥15%) are at least of moderate body size (Fig. 3), but only a third of these are icon tourism attractions.

Some of these species are already at particular risk since they survive only at a single site in one country. Indeed, this is one factor considered by IUCN in the allocation of CR or EN redlisting status. For the 90 species assessed here, 27% survive in only a single population, and reliance on tourism revenue is proportionately higher for more severely threatened species which occur in fewer remaining subpopulations (Fig. 4). Mindoro dwarf buffalo *Bubalus mindorensis,* for example, are protected in only one Philippine national park, with 53% of funding from tourism.

If each individual of each endangered (EN) or critically endangered (CR) mammal species is given equal weight, so that, e.g., one Gilbert’s potoroo is counted the same as one hippopotamus, then in aggregate, tourism protects 4.9% of the 40 EN and 9.6% of the 26 CR mammal species with data available. That is, reliance on tourism revenue is twice as severe for critically endangered as for endangered species.

## Discussion

Our estimates of T are conservative for all species listed, for two main reasons. Firstly, we used maximum published estimates for G. The degree of underestimation from this factor, for different species a, depends on the range of different estimates for G_a_. Secondly, we calculated T_a_ by dividing Σ_i_^n^ S_ia_R_ia_ for the n subpopulations where S and R data are available for each species a, by the global totals G_a_ which also include subpopulations without such data. Thus for the common hippopotamus, as noted earlier, Σ_i_^n^ S_ia_R_ia_ is calculated for 4 subpopulations, but G_a_ is for at least 138. We do not extrapolate from subpopulations with data on S and R to those without, because parks budget structures differ greatly between nations. If parks budgets were available for all African nations, for example, T estimates for African elephant would be increased. The degree of underestimation from this factor depends on the completeness of IUCN subpopulation data for each species, the numbers of subpopulations where it is known to occur, and the countries in which those subpopulations occur.

Arguably, the conservation values of one living individual of different threatened species are not equal, but inversely proportional to total remaining global populations. We could calculate a more complex conservation currency where individuals of different species are weighted according to relative rarity, but this would be less robust than the simpler metric adopted here. Alternatively, we could potentially use a probabilistic rather than an accounting model, calculating how support from tourism increases survival probabilities for each subpopulation, and thus for the species overall. This is not yet feasible, because of uncertainties over raw data [28], controversy over minimum viable population sizes [29], improvements in captive breeding and relocation [30], and rapid changes in contributions of tourism to parks revenues. We found no correlation between T and the recently-developed SAFE index [27], which examines the relationship between minimum viable populations and global population sizes for different species.

This analysis focuses on public protected areas, because these are the most significant conservation reserves for most IUCN-redlisted mammal species. Conservation on other land tenures, however [12], [19], [29], [31], though complex and contested [31]–[33] is also increasingly important for many threatened species, especially as they face additional risks associated with climate change [11], [17], [34]–[36]. Tourism does also contribute to conservation of threatened species on private and community reserves [37]. Subpopulation sizes on these land tenures, however, are generally far smaller than in public protected areas. The results presented here show that revenue from tourism to public parks is currently far more significant for conservation of threatened species globally.

With few exceptions [37], this revenue is raised almost entirely from individual park visitor fees, not commercial tour operators. In countries such as the Philippines, Kenya, Botswana and Zambia, over half of parks funding is from visitors. These proportions are high not only because these parks are popular with tourists, but because government funding for parks agencies in these countries is low. This reflects a new but powerful trend in conservation finance [16]–[18]. For those national parks agencies which do not currently charge high visitor fees, but whose parks protect mammal species in high demand from tourists, agencies are under pressure to raise fees to boost conservation funding. At the same time, governments are imposing new taxes on park-based wildlife tourism. This is currently under intense debate in India and several African nations. This increases the conservation risks identified here. In addition, if tourist demand is weak or access is poor, raising *per capita* fees decreases visitor numbers and revenues.

Rather than relying on tourism, a safer and more effective strategy for conservation of threatened mammal species in impoverished nations would be for international donors to fund park ranger salaries and equipment directly. For species with only a few small subpopulations remaining, funding may also be required for captive breeding and translocations. Tourism does contribute to these [37], but only in a few cases. By funding parks agencies in developing nations directly, wealthier nations can reduce the developing nations’ dependence on tourism. Political pride and patronage in recipient nations currently present barriers to such direct earmarked funding. These barriers could be overcome, however, by linking funding to payments for ecosystem services, including carbon sequestration.

From a research perspective, this contribution answers the many recent calls [1]–[2], [19], [38]–[39] to improve information flows between conservation science and conservation policy. Future evaluations will be more comprehensive, if further data become available; and more accurate, if contributions can be measured using probabilities of species survival rather than number of individuals currently surviving.

## Supporting Information

Table S1 **Subpopulation data for red-listed mammal species in** **Table 1** **.**
(DOC)

Table S2 **Proportions of tourism revenue in protected area budgets, by country.**
(DOC)
