# Supplementary material for: A Population Accounting Approach to Assess Tourism Contributions to Conservation of IUCN-Redlisted Mammal Species
Source: PLoS One. 2012 Sep 12;7(9):e44134. doi: 10.1371/journal.pone.0044134 (PMC3440393; doi:10.1371/journal.pone.0044134)
Supplement: Table S1 — Subpopulation data for red-listed mammal species in Table 1 . (DOC) [file pone.0044134.s001.doc]

Supplementary Table 1: Subpopulation data for threatened mammal species for which the global population size is known and where data on tourism revenue from each country can be estimated.

| ***Species*** | **Common name** | **IUCN** | **Global population (G)** | **Country** | **Reserve name** | **Population estimate (S)** | **Proportion Tourism Revenue (R)** | **Numbers protected by tourism (SR)** |
| --- | --- | --- | --- | --- | --- | --- | --- | --- |
| *Acinonyx jubatus* | Cheetah | VU | 7000-10000 | South Africa | Shamwari Game Reserve | 10 | 47.2 | 5 |
| *Acinonyx jubatus* | Cheetah | VU | 7000-10000 | Tanzania | Loliondo Game Ranch | 26 | 36.7 | 10 |
| *Acinonyx jubatus* | Cheetah | VU | 7000-10000 | Tanzania | Ngorongoro Crater | 28 | 36.7 | 10 |
| *Acinonyx jubatus* | Cheetah | VU | 7000-10000 | South Africa | Hluhluwe Umfolozi National Park | 20 | 47.2 | 9 |
| *Acinonyx jubatus* | Cheetah | VU | 7000-10000 | South Africa | Mountain Zebra National Park | 20 | 47.2 | 9 |
| *Acinonyx jubatus* | Cheetah | VU | 7000-10000 | Tanzania | Simanjiro Game Ranch | 45 | 36.7 | 16 |
| *Acinonyx jubatus* | Cheetah | VU | 7000-10000 | Namibia | Tsavo National Park | 250 | 8.9 | 22 |
| *Acinonyx jubatus* | Cheetah | VU | 7000-10000 | Tanzania | Selous National Park | 65 | 36.7 | 24 |
| *Acinonyx jubatus* | Cheetah | VU | 7000-10000 | Namibia | Etosha National Park | 300 | 8.9 | 27 |
| *Acinonyx jubatus* | Cheetah | VU | 7000-10000 | Botswana | Okavango Delta | 52 | 81.1 | 42 |
| *Acinonyx jubatus* | Cheetah | VU | 7000-10000 | South Africa | Kruger National Park | 200 | 47.2 | 94 |
| *Acinonyx jubatus* | Cheetah | VU | 7000-10000 | Tanzania | Serengeti National Park | 300 | 36.7 | 110 |
| *Acinonyx jubatus* | Cheetah | VU | 7000-10000 | Botswana / South Africa | Kgalagadi Transfrontier Park | 230 | 81.1 | 186 |
| *Ailurus fulgens* | Red Panda | VU | ~ 10000 | India | Singhalila National Park | 46 | 8.0 | 4 |
| *Ailurus fulgens* | Red Panda | VU | ~ 10000 | Nepal | Langtang National Park | 479 | 35.6 | 171 |
| *Ammospermophilus nelsoni* | San Joaquin Antelope Squirrel | EN | 124000-413000 | United States | Carrizo Plains National Monument | 151800 | 7.4 | 11179 |
| *Axis calamianensis* | Calamian Hog Deer | EN | < 1000 | Philippines | Calauit Island Game Preserve and Wildlife Sanctuary | 600 | 53.0 | 318 |
| *Beatragus hunteri* | Hirola | CR | ~ 600 | Kenya | Tsavo East National Park | 105 | 66.1 | 69 |
| *Beatragus hunteri* | Hirola | CR | ~ 600 | Kenya | Ishaqbini Hirola Conservancy | 150 | 66.1 | 99 |
| *Bettongia penicillata* | Woylie/Brush tailed Bettong | CR | <7000 | Australia | Lincoln National Park | 35 | 9.4 | 3 |
| *Bettongia penicillata* | Woylie/Brush tailed Bettong | CR | <7000 | Australia | Venus Bay Island | 35 | 9.4 | 3 |
| *Bettongia penicillata* | Woylie/Brush tailed Bettong | CR | <7000 | Australia | Boyagin Nature Reserve | 100 | 9.4 | 9 |
| *Bettongia penicillata* | Woylie/Brush tailed Bettong | CR | <7000 | Australia | Tutanning Nature Reserve | 100 | 9.4 | 9 |
| *Bettongia penicillata* | Woylie/Brush tailed Bettong | CR | <7000 | Australia | Francois Peron National Park | 100 | 9.4 | 9 |
| *Bettongia penicillata* | Woylie/Brush tailed Bettong | CR | <7000 | Australia | Venus Bay Peninsula Conservation Park | 1500 | 9.4 | 142 |
| *Bettongia penicillata* | Woylie/Brush tailed Bettong | CR | <7000 | Australia | St Peter Island Conservation Park | 2000 | 9.4 | 189 |
| *Blastocerus dichotomus* | Marsh Deer | VU | ~ 45000 | Bolivia | Madidi National Park | 700 | 8.1 | 57 |
| *Blastocerus dichotomus* | Marsh Deer | VU | ~ 45000 | Argentina | Ibera marshes | 2000 | 26.5 | 530 |
| *Brachyteles hypoxanthus* | Northern muriqui | CR | 855 | Brazil | PE Ibitipoc | 7 | 7.8 | 1 |
| *Brachyteles hypoxanthus* | Northern muriqui | CR | 855 | Brazil | PANA Caparaó | 82 | 7.8 | 6 |
| *Brachyteles hypoxanthus* | Northern muriqui | CR | 855 | Brazil | PE Rio Doce | 124 | 7.8 | 10 |
| *Brachyteles hypoxanthus* | Northern muriqui | CR | 855 | Brazil | PE Serra do Brigadeiro | 226 | 7.8 | 18 |
| *Bradypus pygmaeus* | Pygmy Three-toed Sloth | CR | < 5000 | Panama | Comarca Indigenous Reserve, Isla Escudo de Veraguas | 5000 | 13.1 | 655 |
| *Bubalus mindorensis* | Mindoro Dwarf Buffalo | CR | < 250 | Philippines | Mt Iglit-Baco National Park, Mindoro Island | 220 | 53.0 | 117 |
| *Burramys parvus* | Mountain Pygmy Possum | CR | 2250 | Australia | Alpine National Park | 2250 | 9.4 | 213 |
| *Canis rufus* | Red Wolf | CR | < 150 | United States | Alligator River National Wildlife Refuge | 11 | 7.4 | 1 |
| *Canis rufus* | Red Wolf | CR | < 150 | United States | Pocosin Lakes National Wildlife Refuge | 11 | 7.4 | 1 |
| *Canis rufus* | Red Wolf | CR | < 150 | United States | Mattamuskeet National Wildlife Refuge | 11 | 7.4 | 1 |
| *Cephalophus adersi* | Aders' Duiker | CR | < 1000 | Kenya | Arabuko-Sokoko Forest / Dodori National Reserve | 500 | 66.1 | 330 |
| *Cephalophus adersi* | Aders' Duiker | CR | < 1000 | Tanzania | Zanzibar protected areas | 660 | 36.7 | 242 |
| *Cephalophus spadix* | Abbott's Duiker | EN | < 1500 | Tanzania | Livingstone Forest, Kitulo National Park | 40 | 36.7 | 15 |
| *Cephalophus spadix* | Abbott's Duiker | EN | < 1500 | Tanzania | Rubeho Mountains | 50 | 36.7 | 18 |
| *Cercocebus galeritus* | Tana River Crested Mangabey | EN | ~ 1200 | Kenya | Government Land | 500 | 66.1 | 330 |
| *Cercocebus galeritus* | Tana River Crested Mangabey | EN | ~ 1200 | Kenya | Tana River Primate Reserve | 700 | 66.1 | 463 |
| *Cercocebus sanjei* | Sanje Mangabey | EN | < 1300 | Tanzania | Udzungwa Scarp Forest Reserve | 500 | 36.7 | 183 |
| *Cercocebus sanjei* | Sanje Mangabey | EN | < 1300 | Tanzania | Udzungwa Mountains National Park | 800 | 36.7 | 293 |
| *Crypytoprocta ferox* | Fossa | VU | < 2500 | Madagascar | Ankarafantsika National Park | 270 | 5.0 | 13 |
| *Crypytoprocta ferox* | Fossa | VU | < 2500 | Madagascar | Masoala National Park | 414 | 5.0 | 21 |
| *Cynomys parvidens* | Utah Prairie Dog | EN | 8000 | United States | USA reserves | 2560 | 7.4 | 189 |
| *Diceros bicornis* | Black Rhinoceros | CR | 4880 | Namibia | Namibian formal protected areas | 1230 | 8.9 | 110 |
| *Diceros bicornis* | Black Rhinoceros | CR | 4880 | Tanzania | Tanzanian formal protected areas | 79 | 36.7 | 29 |
| *Diceros bicornis* | Black Rhinoceros | CR | 4880 | Kenya | Kenyan formal protected areas | 417 | 66.1 | 276 |
| *Diceros bicornis* | Black Rhinoceros | CR | 4880 | South Africa | South African formal protected areas | 1355 | 47.2 | 640 |
| *Diceros bicornis* | Black Rhinoceros | CR | 4880 | Zambia | Zambian formal protected areas | 27 | 48.3 | 13 |
| *Dipodomys insularis* | San Jose Island Kangaroo Rat | CR | ~ 100 | Mexico | San Jose Island | 100 | 5.9 | 6 |
| *Elephas maximus* | Asian Elephant | EN | 41410–52345 | Nepal | Nepalese reserves | 125 | 35.6 | 45 |
| *Elephas maximus* | Asian Elephant | EN | 41410–52345 | Thailand | Thai Reserves | 3200 | 24.6 | 788 |
| *Elephas maximus* | Asian Elephant | EN | 41410–52345 | India | India | 30770 | 8.0 | 2462 |
| *Equus grevyi* | Grevy's Zebra | EN | 1966-2447 | Kenya | Sibiloi National Park | 22 | 66.1 | 15 |
| *Equus grevyi* | Grevy's Zebra | EN | 1966-2447 | Kenya | Buffalo Springs | 150 | 66.1 | 99 |
| *Equus grevyi* | Grevy's Zebra | EN | 1966-2447 | Kenya | Tsavo East National Park | 200 | 66.1 | 132 |
| *Equus grevyi* | Grevy's Zebra | EN | 1966-2447 | Kenya | Lewa Wildlife Conservancy | 370 | 66.1 | 245 |
| *Equus hemionus* | Asiatic Wild Ass | EN | ~ 24000 | India | Indian Wild Ass Sanctuary | 4000 | 8.0 | 320 |
| *Equus zebra* | Cape Mountain Zebra | VU | ~ 3000 | South Africa | Gamka Mountain Nature Reserve | 20 | 47.2 | 9 |
| *Equus zebra* | Cape Mountain Zebra | VU | ~ 3000 | South Africa | De Hoop Nature Reserve | 65 | 47.2 | 31 |
| *Equus zebra* | Cape Mountain Zebra | VU | ~ 3000 | South Africa | Karoo National Park | 491 | 47.2 | 232 |
| *Equus zebra* | Cape Mountain Zebra | VU | ~ 3000 | South Africa | Mountain Zebra National Park | 596 | 47.2 | 282 |
| *Equus zebra* | Hartmann' Mountain Zebra | VU | ~ 25000 | South Africa | Richtersveld National Park | 15 | 47.2 | 7 |
| *Equus zebra* | Hartmann' Mountain Zebra | VU | ~ 25000 | South Africa | Goegap Nature Reserve | 30 | 47.2 | 14 |
| *Equus zebra* | Hartmann' Mountain Zebra | VU | ~ 25000 | South Africa | Augrabies Falls National Park | 40 | 47.2 | 19 |
| *Equus zebra* | Hartmann' Mountain Zebra | VU | 25000 | Namibia | Namib-Naukluft National Park | 2400 | 8.9 | 214 |
| *Equus zebra* | Hartmann' Mountain Zebra | VU | 25000 | Namibia | Kunene region conservancies | 6400 | 8.9 | 570 |
| *Eulemur cinereiceps* | White-collared Lemur | EN | ~ 7265 | Madagascar | Manombo Special Reserve, Mahabo SR | 750 | 5.0 | 37 |
| *Galidictis grandidieri* | Giant-striped Mongoose | EN | 2650 - 3540 | Madagascar | Tsimanampetsotse National Park | 826 | 5.0 | 41 |
| *Gymnobelideus leadbeateri* | Leadbeater's Possum | EN | 2000 | Australia | Yellingbo Conservation Nature Reserve | 200 | 9.4 | 19 |
| *Hapalemur aureus* | Golden Bamboo Lemur | EN | ~ 1500 | Madagascar | Andringitra National Park | 306 | 5.0 | 15 |
| *Hapalemur aureus* | Golden Bamboo Lemur | EN | ~ 1500 | Madagascar | Ranomafana National Park | 887 | 5.0 | 44 |
| *Hippocamelus bisulcus* | Patagonian Huemul | EN | 1500 | Argentina | Argentinian Reserves | 500 | 26.5 | 133 |
| *Hippocamelus bisulcus* | Patagonian Huemul | EN | 1500 | Chile | Chilean Reserves | 1000 | 37.9 | 379 |
| *Hippopotamus amphibius* | Common Hippopotamus | VU | 125000-148000 | Botswana | Okavango, Chobe, Linyanti Reserves | 1600 | 81.1 | 1297 |
| *Hippopotamus amphibius* | Common Hippopotamus | VU | 125000-148000 | South Africa | Kruger National Park | 2761 | 47.2 | 1304 |
| *Hippopotamus amphibius* | Common Hippopotamus | VU | 125000-148000 | Tanzania | Selous Game Reserve | 20589 | 36.7 | 7546 |
| *Hippopotamus amphibius* | Common Hippopotamus | VU | 125000-148000 | Zambia | Zambia, Luangwa Valley | 22500 | 48.3 | 10868 |
| *Isoodon auratus* | Golden Bandicoot | VU | > 25000 | Australia | Middle Island Nature Reserve | 1000 | 9.4 | 94 |
| *Isoodon auratus barrowensis* | Golden Bandicoot | VU | > 25000 | Australia | Barrow Island Nature Reserve | 20000 | 9.4 | 1889 |
| *Lagorchestes hirsutus bernieri* | Rufous Hare-wallaby | VU | ~ 6000 | Australia | Bernier and Dorre Island Nature Reserve | 2500 | 9.4 | 236 |
| *Lagorchestes hirsutus dorreae* | Rufous Hare-wallaby | VU | ~ 6000 | Australia | Bernier and Dorre Island Nature Reserve | 2500 | 9.4 | 236 |
| *Lagorchestes hirsutus ssp* | Rufous Hare-wallaby | VU | ~ 6000 | Australia | Montebello Islands Marine Park | 120 | 9.4 | 11 |
| *Lagostrophus fasciatus* | Banded Hare Wallaby | EN | < 10000 | Australia | Bernier and Dorre Island Nature Reserve | 4500 | 9.4 | 425 |
| *Lagostrophus fasciatus* | Banded Hare Wallaby | EN | < 10000 | Australia | Faure Island Sanctuary | 4500 | 9.4 | 425 |
| *Lasiorhinus krefftii* | Northern Hairy-nosed Wombat | CR | 115 | Australia | Epping Forest National Park | 115 | 9.4 | 11 |
| *Leontopithecus caissara* | Black-faced Lion tamarin | CR | 400 | Brazil | PANA do Superagui | 183 | 7.8 | 14 |
| *Leontopithecus chrysomelas* | Golden-headed lion tamarin | EN | 6000 -15000 | Brazil | EE Lemos Maia | 54 | 7.8 | 4 |
| *Leontopithecus chrysomelas* | Golden-headed lion tamarin | EN | 6000 -15000 | Brazil | EE Canavieiras | 55 | 7.8 | 4 |
| *Leontopithecus chrysomelas* | Golden-headed lion tamarin | EN | 6000 -15000 | Brazil | REBIO Una | 425 | 7.8 | 33 |
| *Leontopithecus chrysomelas* | Golden-headed lion tamarin | EN | 6000 -15000 | Brazil | PANA Serra das Lontras | 1848 | 7.8 | 144 |
| *Leontopithecus chrysomelas* | Golden-headed lion tamarin | EN | 6000 -15000 | Brazil | RVS Una | 2530 | 7.8 | 197 |
| *Leontopithecus chrysopygus* | Black lion tamarin | EN | 1000 | Brazil | PE Morro do Diabo | 820 | 7.8 | 64 |
| *Leontopithecus rosalia* | Golden lion tamarin | EN | 1000 | Brazil | REBIO União | 130 | 7.8 | 10 |
| *Leontopithecus rosalia* | Golden lion tamarin | EN | 1000 | Brazil | REBIO Una | 246 | 7.8 | 19 |
| *Leontopithecus rosalia* | Golden lion tamarin | EN | 1000 | Brazil | REBIO Poço das Antas | 660 | 7.8 | 51 |
| *Leontopithecus rosalia* | Golden lion tamarin | EN | 1000 | Brazil | Natural CP Taquara | 1069 | 7.8 | 83 |
| *Leporillus conditor* | Greater Stick-nest Rat | VU | 4000 | Australia | Heirisson Prong Conservation Reserve | 10 | 9.4 | 1 |
| *Leporillus conditor* | Greater Stick-nest Rat | VU | 4000 | Australia | Roxby Downs, Arid Recovery | 300 | 9.4 | 28 |
| *Leporillus conditor* | Greater Stick-nest Rat | VU | 4000 | Australia | Nuyts Archipelago Conservation Park | 1000 | 9.4 | 94 |
| *Loxodonta africana* | African Elephant | VU | ~ 500000 | Namibia | Kunene region conservancies | 210 | 8.9 | 19 |
| *Loxodonta africana* | African Elephant | VU | ~ 500000 | South Africa | Shamwari Game Reserve | 61 | 47.2 | 29 |
| *Loxodonta africana* | African Elephant | VU | ~ 500000 | South Africa | Manyeleti Game Reserve | 71 | 47.2 | 34 |
| *Loxodonta africana* | African Elephant | VU | ~ 500000 | South Africa | Makalali Game Reserve | 72 | 47.2 | 34 |
| *Loxodonta africana* | African Elephant | VU | ~ 500000 | South Africa | Selati Game Reserve | 85 | 47.2 | 40 |
| *Loxodonta africana* | African Elephant | VU | ~ 500000 | South Africa | Welgevonden Game Reserve / Marakele National Park | 100 | 47.2 | 47 |
| *Loxodonta africana* | African Elephant | VU | ~ 500000 | Zambia | Chisome Game Area | 128 | 48.3 | 62 |
| *Loxodonta africana* | African Elephant | VU | ~ 500000 | South Africa | Pilanesberg National Park | 140 | 47.2 | 66 |
| *Loxodonta africana* | African Elephant | VU | ~ 500000 | South Africa | Umbabat Nature Reserve | 163 | 47.2 | 77 |
| *Loxodonta africana* | African Elephant | VU | ~ 500000 | Namibia | Nyae Nyae Conservancy | 967 | 8.9 | 86 |
| *Loxodonta africana* | African Elephant | VU | ~ 500000 | South Africa | Hluhluwe Umfolozi National Park | 346 | 47.2 | 163 |
| *Loxodonta africana* | African Elephant | VU | ~ 500000 | Namibia | Etosha National Park | 2057 | 8.9 | 183 |
| *Loxodonta africana* | African Elephant | VU | ~ 500000 | Zambia | Kasonso-Busanga Game Area | 401 | 48.3 | 194 |
| *Loxodonta africana* | African Elephant | VU | ~ 500000 | Tanzania | Inyonga Game Conservation Area | 600 | 36.7 | 220 |
| *Loxodonta africana* | African Elephant | VU | ~ 500000 | South Africa | Madikwe Game Reserve | 455 | 47.2 | 215 |
| *Loxodonta africana* | African Elephant | VU | ~ 500000 | South Africa | Balule Nature Reserve | 457 | 47.2 | 216 |
| *Loxodonta africana* | African Elephant | VU | ~ 500000 | South Africa | Addo Elephant National Park | 459 | 47.2 | 217 |
| *Loxodonta africana* | African Elephant | VU | ~ 500000 | Tanzania | Kilimanjaro National Park | 793 | 36.7 | 291 |
| *Loxodonta africana* | African Elephant | VU | ~ 500000 | South Africa | Klaserie Nature Reserve | 569 | 47.2 | 269 |
| *Loxodonta africana* | African Elephant | VU | ~ 500000 | South Africa | Limpopo National Park | 630 | 47.2 | 298 |
| *Loxodonta africana* | African Elephant | VU | ~ 500000 | Namibia | Khaudom-Kavango | 3787 | 8.9 | 337 |
| *Loxodonta africana* | African Elephant | VU | ~ 500000 | South Africa | Timbavati Nature Reserve | 712 | 47.2 | 336 |
| *Loxodonta africana* | African Elephant | VU | ~ 500000 | Tanzania | Tarangire National Park | 1119 | 36.7 | 410 |
| *Loxodonta africana* | African Elephant | VU | ~ 500000 | South Africa | Sabie Sands Game Reserve | 857 | 47.2 | 405 |
| *Loxodonta africana* | African Elephant | VU | ~ 500000 | Zambia | Luawata Hunting Block | 968 | 48.3 | 468 |
| *Loxodonta africana* | African Elephant | VU | ~ 500000 | Zambia | Lupande Game Management Area | 975 | 48.3 | 471 |
| *Loxodonta africana* | African Elephant | VU | ~ 500000 | Zambia | Musalangu Game Area | 1011 | 48.3 | 488 |
| *Loxodonta africana* | African Elephant | VU | ~ 500000 | Tanzania | Serengeti National Park | 1472 | 36.7 | 540 |
| *Loxodonta africana* | African Elephant | VU | ~ 500000 | Kenya | Mau Forest Complex | 1003 | 66.1 | 663 |
| *Loxodonta africana* | African Elephant | VU | ~ 500000 | Zambia | Lower Zambezi National Park | 1477 | 48.3 | 713 |
| *Loxodonta africana* | African Elephant | VU | ~ 500000 | Namibia | Caprivi region | 8725 | 8.9 | 777 |
| *Loxodonta africana* | African Elephant | VU | ~ 500000 | Botswana | Tuli Game Reserve | 1038 | 81.1 | 841 |
| *Loxodonta africana* | African Elephant | VU | ~ 500000 | Kenya | Amboseli National Park | 1417 | 66.1 | 936 |
| *Loxodonta africana* | African Elephant | VU | ~ 500000 | Kenya | Maasai Mara Reserve | 1655 | 66.1 | 1094 |
| *Loxodonta africana* | African Elephant | VU | ~ 500000 | Botswana | Nxai Pan & Makgadikgadi National Park | 1436 | 81.1 | 1164 |
| *Loxodonta africana* | African Elephant | VU | ~ 500000 | Kenya | Aberdare National Park | 1840 | 66.1 | 1216 |
| *Loxodonta africana* | African Elephant | VU | ~ 500000 | Tanzania | Katavi National Park | 4102 | 36.7 | 1503 |
| *Loxodonta africana* | African Elephant | VU | ~ 500000 | Tanzania | Ugalla River Game Reserve | 4133 | 36.7 | 1515 |
| *Loxodonta africana* | African Elephant | VU | ~ 500000 | Zambia | North Luangwa National Park | 3235 | 48.3 | 1563 |
| *Loxodonta africana* | African Elephant | VU | ~ 500000 | Kenya | Mt Kenya National Park | 2911 | 66.1 | 1924 |
| *Loxodonta africana* | African Elephant | VU | ~ 500000 | Zambia | South Luangwa National Park | 4459 | 48.3 | 2154 |
| *Loxodonta africana* | African Elephant | VU | ~ 500000 | Zambia | Kafue National Park | 6306 | 48.3 | 3046 |
| *Loxodonta africana* | African Elephant | VU | ~ 500000 | Tanzania | Moyowosi-Kigosi Game Reserve | 9541 | 36.7 | 3497 |
| *Loxodonta africana* | African Elephant | VU | ~ 500000 | Kenya | Laikipia ecosystem | 5447 | 66.1 | 3600 |
| *Loxodonta africana* | African Elephant | VU | ~ 500000 | Kenya | Tsavo National Park | 9021 | 66.1 | 5962 |
| *Loxodonta africana* | African Elephant | VU | ~ 500000 | South Africa | Kruger National Park | 12427 | 47.2 | 5871 |
| *Loxodonta africana* | African Elephant | VU | ~ 500000 | Tanzania | Ruhaha-Rungwa Ecosystem | 35409 | 36.7 | 12978 |
| *Loxodonta africana* | African Elephant | VU | ~ 500000 | Botswana | Okavango Delta | 31191 | 81.1 | 25286 |
| *Loxodonta africana* | African Elephant | VU | ~ 500000 | Tanzania | Selous Ecosystem | 70406 | 36.7 | 25805 |
| *Loxodonta africana* | African Elephant | VU | ~ 500000 | Botswana | Chobe National Park | 40767 | 81.1 | 33049 |
| *Lycaon pictus* | African Wild Dog | EN | 3000-5500 | South Africa | Kwandwe Game Reserve | 5 | 47.2 | 2 |
| *Lycaon pictus* | African Wild Dog | EN | 3000-5500 | Namibia | Caprivi region | 30 | 8.9 | 3 |
| *Lycaon pictus* | African Wild Dog | EN | 3000-5500 | South Africa | Madikwe Game Reserve | 6 | 47.2 | 3 |
| *Lycaon pictus* | African Wild Dog | EN | 3000-5500 | Tanzania | Serengeti National Park | 75 | 36.7 | 27 |
| *Lycaon pictus* | African Wild Dog | EN | 3000-5500 | Botswana | Moremi Reserve | 190 | 81.1 | 154 |
| *Lycaon pictus* | African Wild Dog | EN | 3000-5500 | South Africa | Kruger National Park | 350 | 47.2 | 165 |
| *Lycaon pictus* | African Wild Dog | EN | 3000-5500 | Tanzania | Selous Game Reserve | 900 | 36.7 | 330 |
| *Macaca silenus* | Lion-tailed Macaque | EN | < 4000 | India | Anaimalai Hills, Tamil Nadu - 5 reserves | 500 | 8.0 | 40 |
| *Macaca silenus* | Lion-tailed Macaque | EN | < 4000 | India | Kerala Forest - 10 reserves | 1200 | 8.0 | 96 |
| *Macroderma gigas* | Ghost Bat | VU | 7000-9000 | Australia | Pilbara | 600 | 9.4 | 57 |
| *Macroderma gigas* | Ghost Bat | VU | 7000-9000 | Australia | Wet Tropics World Heritage Area | 1000 | 9.4 | 94 |
| *Macroderma gigas* | Ghost Bat | VU | 7000-9000 | Australia | Northern territory | 3500 | 9.4 | 331 |
| *Macroderma gigas* | Ghost Bat | VU | 7000-9000 | Australia | Kimberley | 4000 | 9.4 | 378 |
| *Macrotis lagotis* | Greater Bilby | VU | <10000 | Australia | Scotia Sanctuary | 40 | 9.4 | 4 |
| *Macrotis lagotis* | Greater Bilby | VU | <10000 | Australia | Currawinya National Park | 50 | 9.4 | 5 |
| *Macrotis lagotis* | Greater Bilby | VU | <10000 | Australia | Venus Bay Conservation Park | 100 | 9.4 | 9 |
| *Macrotis lagotis* | Greater Bilby | VU | <10000 | Australia | Francois Peron National Park | 200 | 9.4 | 19 |
| *Macrotis lagotis* | Greater Bilby | VU | <10000 | Australia | Thistle Island, South Australia | 500 | 9.4 | 47 |
| *Macrotis lagotis* | Greater Bilby | VU | <10000 | Australia | Roxby Downs, Arid Recovery | 500 | 9.4 | 47 |
| *Melursus ursinus* | Sloth Bear | VU | < 20000 | India | 174 Indian protected areas | 8400 | 8.0 | 672 |
| *Mesocapromys angelcabrerai* | Cabrera's Hutia | EN | < 2500 | Cuba | Wildlife Refuge Cayos Anamaria | 2500 | 5.0 | 125 |
| *Mesocapromys auritus* | Eared Hutia | EN | < 2500 | Cuba | Lanzanillo-Pajonal-Fragoso Wildlife Refuge | 2500 | 5.0 | 125 |
| *Mustela nigripes* | Black-footed Ferret | EN | 500-1000 | United States | USA reserves | 608 | 7.4 | 45 |
| *Mustela nigripes* | Black-footed Ferret | EN | 500-1000 | United States | UL Bend National Wildlife Refuge | 13 | 7.4 | 1 |
| *Mustela nigripes* | Black-footed Ferret | EN | 500-1000 | United States | Lower Brule Indian Reservation | 14 | 7.4 | 1 |
| *Mustela nigripes* | Black-footed Ferret | EN | 500-1000 | United States | Badlands National Park | 20 | 7.4 | 1 |
| *Mustela nigripes* | Black-footed Ferret | EN | 500-1000 | United States | Rosebud Indian Reservation | 30 | 7.4 | 2 |
| *Mustela nigripes* | Black-footed Ferret | EN | 500-1000 | United States | Cheyenne River Indian Reservation | 150 | 7.4 | 11 |
| *Myotis sodalis* | Indiana Bat | EN | ~ 400000 | United States | Ryden Cave Conservation Area | 10 | 7.4 | 1 |
| *Myotis sodalis* | Indiana Bat | EN | ~ 400000 | United States | USA reserves | 210125 | 7.4 | 15474 |
| *Myotis sodalis* | Indiana Bat | EN | ~ 400000 | United States | Onyx Cave Conservation Area | 180 | 7.4 | 13 |
| *Myotis sodalis* | Indiana Bat | EN | ~ 400000 | United States | Kingdom Come State Park Nature Preserve | 1844 | 7.4 | 136 |
| *Myotis sodalis* | Indiana Bat | EN | ~ 400000 | United States | Mammoth Cave National Park | 3100 | 7.4 | 228 |
| *Myotis sodalis* | Indiana Bat | EN | ~ 400000 | United States | Great Smoky Mountains National Park | 7861 | 7.4 | 579 |
| *Myotis sodalis* | Indiana Bat | EN | ~ 400000 | United States | Buffalo National River | 9270 | 7.4 | 683 |
| *Myotis sodalis* | Indiana Bat | EN | ~ 400000 | United States | Twin Domes Nature Preserve | 36800 | 7.4 | 2710 |
| *Myotis sodalis* | Indiana Bat | EN | ~ 400000 | United States | Pilot Knob National Wildlife Refuge | 50550 | 7.4 | 3722 |
| *Myotis sodalis* | Indiana Bat | EN | ~ 400000 | United States | O'Bannon Woods State Park | 54913 | 7.4 | 4044 |
| *Myrmecobius fasciatus* | Numbat | EN | < 1000 | Australia | Dryandra Woodland | 550 | 9.4 | 52 |
| *Mysateles meridionalis* | Southern Hutia | CR | < 250 | Cuba | Isla de la Joventud | 250 | 5.0 | 12 |
| *Natalus primus* | Cuban Greater Funnel-eared Bat | CR | ~ 100 | Cuba | Isla de la Joventud | 100 | 5.0 | 5 |
| *Nilgiritragus hylocrius* | Nilgiri Tahr | EN | 2000-2500 | India | Silent Valley National Park | 30 | 8.0 | 2 |
| *Nilgiritragus hylocrius* | Nilgiri Tahr | EN | 2000-2500 | India | Parambikulam Wildlife Sanctuary | 120 | 8.0 | 10 |
| *Nilgiritragus hylocrius* | Nilgiri Tahr | EN | 2000-2500 | India | Eravikulam National Park | 760 | 8.0 | 61 |
| *Nyctimene rabori* | Philippine Tube-nosed Fruit Bat | EN | < 2500 | Philippines | North Negros Forest Reserve | 1500 | 53.0 | 795 |
| *Onychogalea fraenata* | Bridled Nailtail Wallaby | EN | 450 | Australia | Taunton National Park | 125 | 9.4 | 12 |
| *Onychogalea fraenata* | Bridled Nailtail Wallaby | EN | 450 | Australia | Idalia National Park | 125 | 9.4 | 12 |
| *Panthera leo* | Lion | VU | ~ 25000 | Kenya | Aberdare National Park | 7 | 66.1 | 5 |
| *Panthera leo* | Lion | VU | ~ 25000 | South Africa | Eastern Cape reserves | 13 | 47.2 | 6 |
| *Panthera leo* | Lion | VU | ~ 25000 | Tanzania | Manyara National Park | 20 | 36.7 | 7 |
| *Panthera leo* | Lion | VU | ~ 25000 | Kenya | Amboseli National Park | 20 | 66.1 | 13 |
| *Panthera leo* | Lion | VU | ~ 25000 | Kenya | Nairobi National Park | 22 | 66.1 | 15 |
| *Panthera leo* | Lion | VU | ~ 25000 | Kenya | Nakuru National Park | 28 | 66.1 | 19 |
| *Panthera leo* | Lion | VU | ~ 25000 | Tanzania | Ngorongoro Crater | 53 | 36.7 | 19 |
| *Panthera leo* | Lion | VU | ~ 25000 | Namibia | Etosha National Park | 230 | 8.9 | 20 |
| *Panthera leo* | Lion | VU | ~ 25000 | Kenya | Kora National Reserve | 40 | 66.1 | 26 |
| *Panthera leo* | Lion | VU | ~ 25000 | Botswana | Makgadigadi Pans National Park | 39 | 81.1 | 32 |
| *Panthera leo* | Lion | VU | ~ 25000 | Kenya | Meru National Park, Bisanadi Reserve | 80 | 66.1 | 53 |
| *Panthera leo* | Lion | VU | ~ 25000 | South Africa | Madikwe Game Reserve, Pilanesburg National Park | 110 | 47.2 | 52 |
| *Panthera leo* | Lion | VU | ~ 25000 | South Africa | Hluhluwe Umfolozi National Park | 120 | 47.2 | 57 |
| *Panthera leo* | Lion | VU | ~ 25000 | Kenya | Laikipia Ecosystem | 120 | 66.1 | 79 |
| *Panthera leo* | Lion | VU | ~ 25000 | Kenya | Maasai Mara Reserve | 120 | 66.1 | 79 |
| *Panthera leo* | Lion | VU | ~ 25000 | Kenya | Galana Game Ranch | 150 | 66.1 | 99 |
| *Panthera leo* | Lion | VU | ~ 25000 | Botswana | Kwando Reserve | 213 | 81.1 | 173 |
| *Panthera leo* | Lion | VU | ~ 25000 | Botswana | Southern Kgalagadi Wildlife Management Areas | 225 | 81.1 | 182 |
| *Panthera leo* | Lion | VU | ~ 25000 | Botswana | Central Kalahari Game Reserve | 312 | 81.1 | 253 |
| *Panthera leo* | Lion | VU | ~ 25000 | Botswana / South Africa | Kgalagadi Transfrontier Park | 458 | 81.1 | 371 |
| *Panthera leo* | Lion | VU | ~ 25000 | Kenya | Tsavo National Park | 675 | 66.1 | 446 |
| *Panthera leo* | Lion | VU | ~ 25000 | Zambia | Kafue National Park, Lower Zambezi National Park | 1500 | 48.3 | 725 |
| *Panthera leo* | Lion | VU | ~ 25000 | Tanzania | Serengeti National Park | 2500 | 36.7 | 916 |
| *Panthera leo* | Lion | VU | ~ 25000 | South Africa | Kruger National Park | 2200 | 47.2 | 1039 |
| *Panthera leo* | Lion | VU | ~ 25000 | Botswana | Okavango Delta | 1438 | 81.1 | 1166 |
| *Panthera leo* | Lion | VU | ~ 25000 | Tanzania | Selous Game Reserve | 3750 | 36.7 | 1374 |
| *Panthera tigris* | Tiger | EN | 3000-5000 | Nepal | Shuklaphanta Wildlife Reserve | 8 | 35.6 | 3 |
| *Panthera tigris* | Tiger | EN | 3000-5000 | India | Ranthambhore Tiger Reserve | 34 | 8.0 | 3 |
| *Panthera tigris* | Tiger | EN | 3000-5000 | India | Pilibhit Tiger Reserve | 36 | 8.0 | 3 |
| *Panthera tigris* | Tiger | EN | 3000-5000 | India | Bandipur Tiger Reserve | 70 | 8.0 | 6 |
| *Panthera tigris* | Tiger | EN | 3000-5000 | Nepal | Bardia National Park | 18 | 35.6 | 6 |
| *Panthera tigris* | Tiger | EN | 3000-5000 | India | Kaziranga Tiger Reserve | 86 | 8.0 | 7 |
| *Panthera tigris* | Tiger | EN | 3000-5000 | Thailand | Huai Kha Khaeng National Park | 113 | 24.6 | 28 |
| *Panthera tigris* | Tiger | EN | 3000-5000 | Nepal | Chitwan National Park | 91 | 35.6 | 32 |
| *Panthera tigris* | Tiger | EN | 3000-5000 | India | Indian Protected areas (listed below) | 1411 | 8.0 | 113 |
| *Panthera tigris* | Tiger | EN | 3000-5000 | India | Nameri Tiger Reserve |  | 8.0 | 0 |
| *Panthera tigris* | Tiger | EN | 3000-5000 | India | Pakhui Tiger Reserve |  | 8.0 | 0 |
| *Panthera tigris* | Tiger | EN | 3000-5000 | India | Nagarhole Tiger Reserve |  | 8.0 | 0 |
| *Panthera tigris* | Tiger | EN | 3000-5000 | India | Bandhavgarh Tiger Reserve |  | 8.0 | 0 |
| *Panthera tigris* | Tiger | EN | 3000-5000 | India | Kanha Tiger Reserve |  | 8.0 | 0 |
| *Panthera tigris* | Tiger | EN | 3000-5000 | India | Pench Tiger Reserve |  | 8.0 | 0 |
| *Panthera tigris* | Tiger | EN | 3000-5000 | India | Corbett Tiger Reserve |  | 8.0 | 0 |
| *Panthera tigris* | Tiger | EN | 3000-5000 | India | Sunderbans Tiger Reserve |  | 8.0 | 0 |
| *Panthera uncia* | Snow Leopard | EN | 4080-6590 | India | Hemis National Park | 100 | 8.0 | 8 |
| *Panthera uncia* | Snow Leopard | EN | 4080-6590 | Nepal | Nepalese protected areas (listed below) | 500 | 35.6 | 178 |
| *Panthera uncia* | Snow Leopard | EN | 4080-6590 | Nepal | Annapurna Conservation Area |  | 35.6 | 0 |
| *Panthera uncia* | Snow Leopard | EN | 4080-6590 | Nepal | Langtang National Park |  | 35.6 | 0 |
| *Panthera uncia* | Snow Leopard | EN | 4080-6590 | Nepal | Shey-Phoksundo National Park |  | 35.6 | 0 |
| *Panthera uncia* | Snow Leopard | EN | 4080-6590 | India | Indian protected areas | 600 | 8.0 | 48 |
| *Parantechinus apicalis* | Dibbler | EN | 500-1000 | Australia | Escape Island Nature Reserve (Translocated) | 30 | 9.4 | 3 |
| *Parantechinus apicalis* | Dibbler | EN | 500-1000 | Australia | Peniup Nature Reserve (Translocated) | 30 | 9.4 | 3 |
| *Parantechinus apicalis* | Dibbler | EN | 500-1000 | Australia | Stirling Range National Park (Translocated) | 30 | 9.4 | 3 |
| *Parantechinus apicalis* | Dibbler | EN | 500-1000 | Australia | Boullanger Island Nature Reserve | 180 | 9.4 | 17 |
| *Perameles bougainville* | Western Barred Bandicoot | EN | <10000 | Australia | Faure Island Sanctuary | 20 | 9.4 | 2 |
| *Perameles bougainville* | Western Barred Bandicoot | EN | <10000 | Australia | Roxby Downs, Arid Recovery | 40 | 9.4 | 4 |
| *Perameles bougainville* | Western Barred Bandicoot | EN | <10000 | Australia | Heirisson Prong Conservation Reserve | 200 | 9.4 | 19 |
| *Perameles bougainville* | Western Barred Bandicoot | EN | <10000 | Australia | Bernier and Dorre Island Nature Reserve | 5000 | 9.4 | 472 |
| *Phascogale pirata* | Northern Brush-tailed Phascogale | VU | <10000 | Australia | Melville Island | 1000 | 9.4 | 94 |
| *Phascogale pirata* | Northern Brush-tailed Phascogale | VU | <10000 | Australia | Cobourg Peninsula | 1000 | 9.4 | 94 |
| *Phascogale pirata* | Northern Brush-tailed Phascogale | VU | <10000 | Australia | West Pellew Island | 1000 | 9.4 | 94 |
| *Phascogale pirata* | Northern Brush-tailed Phascogale | VU | <10000 | Australia | Kakadu National Park | 1000 | 9.4 | 94 |
| *Phascogale pirata* | Northern Brush-tailed Phascogale | VU | <10000 | Australia | Garig Gunak Barlu National Park | 1000 | 9.4 | 94 |
| *Phascogale pirata* | Northern Brush-tailed Phascogale | VU | <10000 | Australia | Litchfield National Park | 1000 | 9.4 | 94 |
| *Porcula salvania* | Pygmy Hog | CR | < 500 | India | Manas National Park | 300 | 8.0 | 24 |
| *Potorous gilbertii* | Gilbert's Potoroo | CR | 40 | Australia | Two Peoples Bay Nature Reserve | 40 | 9.4 | 4 |
| *Procolobus gordonorum* | Udzungwa Red Colobus | EN | 10000-15400 | Tanzania | Magombera Forest Reserve | 1000 | 36.7 | 367 |
| *Procolobus kirkii* | Zanzibar Red Colobus | EN | < 2000 | Tanzania | Jozani-Chwaka Bay National Park | 500 | 36.7 | 183 |
| *Procyon pygmaeus* | Pygmy raccoon | CR | < 1000 | Mexico | Cozumel Island | 1000 | 5.9 | 59 |
| *Prolemur simus* | Greater Bamboo Lemur | CR | < 100 | Madagascar | Ranomafana National Park | 20 | 5.0 | 1 |
| *Propithecus candidus* | Silky Sifaka | CR | < 250 | Madagascar | Marojejy National Park | 12 | 5.0 | 1 |
| *Propithecus perrieri* | Perrier’s Sifaka | CR | < 250 | Madagascar | Analamerana Special Reserve | 230 | 5.0 | 11 |
| *Propithecus tattersalli* | Golden-crowned Sifaka | EN | 6000-10000 | Madagascar | Daraina region | 4600 | 5.0 | 229 |
| *Pseudalopex fulvipes* | Darwin's Fox | CR | < 250 | Chile | Nahuelbuta National Park | 75 | 37.9 | 28 |
| *Pseudomys fieldi* | Shark Bay Mouse | VU | 2000 | Australia | Faure Island Sanctuary | 200 | 9.4 | 19 |
| *Pseudomys fieldi* | Shark Bay Mouse | VU | 2000 | Australia | Bernier and Dorre Island Nature Reserve | 300 | 9.4 | 28 |
| *Pseudomys fieldi* | Shark Bay Mouse | VU | 2000 | Australia | North West Island | 1000 | 9.4 | 94 |
| *Pseudomys fumeus* | Smoky Mouse | EN | < 2500 | Australia | Kosciuszko National Park | 250 | 9.4 | 24 |
| *Pseudomys fumeus* | Smoky Mouse | EN | < 2500 | Australia | Namadgi National Park | 250 | 9.4 | 24 |
| *Pseudomys fumeus* | Smoky Mouse | EN | < 2500 | Australia | South East Forests National Park | 250 | 9.4 | 24 |
| *Pseudomys novaehollandiae* | New Holland Mouse | VU | < 10000 | Australia | Angelsea Flora Reserve | 1000 | 9.4 | 94 |
| *Pseudomys novaehollandiae* | New Holland Mouse | VU | < 10000 | Australia | Providence Ponds Flora and Fauna Reserve | 1000 | 9.4 | 94 |
| *Pseudomys novaehollandiae* | New Holland Mouse | VU | < 10000 | Australia | Gippsland Lakes Coastal Park | 1000 | 9.4 | 94 |
| *Pseudomys novaehollandiae* | New Holland Mouse | VU | < 10000 | Australia | Wilsons Promontory National Park | 1000 | 9.4 | 94 |
| *Pseudomys novaehollandiae* | New Holland Mouse | VU | < 10000 | Australia | Freycinet National Park | 1000 | 9.4 | 94 |
| *Pseudomys novaehollandiae* | New Holland Mouse | VU | < 10000 | Australia | Mt William National Park | 1000 | 9.4 | 94 |
| *Pseudomys novaehollandiae* | New Holland Mouse | VU | < 10000 | Australia | Coles Bay Conservation Area | 1000 | 9.4 | 94 |
| *Pseudomys novaehollandiae* | New Holland Mouse | VU | < 10000 | Australia | Bay of Fires Conservation Area | 1000 | 9.4 | 94 |
| *Pseudomys novaehollandiae* | New Holland Mouse | VU | < 10000 | Australia | Darling Range Conservation Area | 1000 | 9.4 | 94 |
| *Pseudomys novaehollandiae* | New Holland Mouse | VU | < 10000 | Australia | Wyrrabalong National Park | 1000 | 9.4 | 94 |
| *Pseudomys novaehollandiae* | New Holland Mouse | VU | < 10000 | Australia | Yuraygir National Park | 1000 | 9.4 | 94 |
| *Pseudomys novaehollandiae* | New Holland Mouse | VU | < 10000 | Australia | Wallingat National Park | 1000 | 9.4 | 94 |
| *Pseudomys oralis* | Hastings River Mouse | VU | ~ 10000 | Australia | Barrington Tops National Park | 1000 | 9.4 | 94 |
| *Pseudomys oralis* | Hastings River Mouse | VU | ~ 10000 | Australia | Oxley Wild Rivers National Park | 1000 | 9.4 | 94 |
| *Pseudomys oralis* | Hastings River Mouse | VU | ~ 10000 | Australia | Werrikimbe National Park | 1000 | 9.4 | 94 |
| *Pseudomys oralis* | Hastings River Mouse | VU | ~ 10000 | Australia | Blicks River Flora Reserve | 1000 | 9.4 | 94 |
| *Pseudomys oralis* | Hastings River Mouse | VU | ~ 10000 | Australia | Edwards Plain Flora Reserve | 1000 | 9.4 | 94 |
| *Pseudomys oralis* | Hastings River Mouse | VU | ~ 10000 | Australia | Chaelundi National Park | 1000 | 9.4 | 94 |
| *Pseudomys oralis* | Hastings River Mouse | VU | ~ 10000 | Australia | Deua National Park | 1000 | 9.4 | 94 |
| *Pseudomys oralis* | Hastings River Mouse | VU | ~ 10000 | Australia | Mount Royal National Park | 1000 | 9.4 | 94 |
| *Pseudomys oralis* | Hastings River Mouse | VU | ~ 10000 | Australia | Nymboida National Park | 1000 | 9.4 | 94 |
| *Rhinoceros unicornis* | Greater One-horned Rhino | VU | 2575 | India | Manas National Park | 3 | 8.0 | 0 |
| *Rhinoceros unicornis* | Greater One-horned Rhino | VU | 2575 | India | Dudhwa National Park | 21 | 8.0 | 2 |
| *Rhinoceros unicornis* | Greater One-horned Rhino | VU | 2575 | Nepal | Suklaphanta National Park | 6 | 35.6 | 2 |
| *Rhinoceros unicornis* | Greater One-horned Rhino | VU | 2575 | India | Gorumara National Park | 27 | 8.0 | 2 |
| *Rhinoceros unicornis* | Greater One-horned Rhino | VU | 2575 | India | Orang National Park | 68 | 8.0 | 5 |
| *Rhinoceros unicornis* | Greater One-horned Rhino | VU | 2575 | India | Pabitora National Park | 81 | 8.0 | 6 |
| *Rhinoceros unicornis* | Greater One-horned Rhino | VU | 2575 | India | Jaldapara National Park | 108 | 8.0 | 9 |
| *Rhinoceros unicornis* | Greater One-horned Rhino | VU | 2575 | Nepal | Bardia National Park | 35 | 35.6 | 12 |
| *Rhinoceros unicornis* | Greater One-horned Rhino | VU | 2575 | Nepal | Chitwan National Park | 372 | 35.6 | 132 |
| *Rhinoceros unicornis* | Greater One-horned Rhino | VU | 2575 | India | Kaziranga National Park | 1855 | 8.0 | 148 |
| *Rhynchocyon udzungwensis* | Grey-faced Sengi | VU | 15000-24000 | Tanzania | Udzungwa Mountains National Park, Kilombero NR | 24000 | 36.7 | 8797 |
| *Romerolagus diazi* | Volcano Rabbit | EN | 2478-12120 | Mexico | Izta-Popo Zoquiapan National Park | 2478 | 5.9 | 146 |
| *Rucervus duvaucelii* | Barasingha, Swamp Deer | VU | 3500-5100 | India | Lagga Bagga Protected Area | 300 | 8.0 | 24 |
| *Rucervus duvaucelii* | Barasingha, Swamp Deer | VU | 3500-5100 | India | Kanha Tiger Reserve | 500 | 8.0 | 40 |
| *Rucervus duvaucelii* | Barasingha, Swamp Deer | VU | 3500-5100 | India | Indian protected areas | 2400 | 8.0 | 192 |
| *Rucervus duvaucelii* | Barasingha, Swamp Deer | VU | 3500-5100 | Nepal | Nepalese protected areas | 1800 | 35.6 | 641 |
| *Rungwecebus kipunji* | Kipunji | CR | ~ 1000 | Tanzania | Kilombero Nature Reserve | 75 | 36.7 | 27 |
| *Rungwecebus kipunji* | Kipunji | CR | ~ 1000 | Tanzania | Mt Rungwe Catchment Forest Reserve | 502 | 36.7 | 184 |
| *Rungwecebus kipunji* | Kipunji | CR | ~ 1000 | Tanzania | Kitulo National Park | 540 | 36.7 | 198 |
| *Saguinus oedipus* | Cotton-headed Tamarin | CR | ~ 6000 | Colombia | Paramillo National Park | 2459 | 7.6 | 187 |
| *Sarcophilus harrisii* | Tasmanian Devil | EN | 10000-25000 | Australia | Tasmanian protected areas | 7000 | 9.4 | 661 |
| *Setonix brachyurus* | Quokka | VU | < 10000 | Australia | Mount Manypeaks Nature Reserve | 50 | 9.4 | 5 |
| *Setonix brachyurus* | Quokka | VU | < 10000 | Australia | Stirling Range National Park | 50 | 9.4 | 5 |
| *Setonix brachyurus* | Quokka | VU | < 10000 | Australia | Tinkelelup Nature Reserve | 50 | 9.4 | 5 |
| *Setonix brachyurus* | Quokka | VU | < 10000 | Australia | Central Jarrah State Forest | 100 | 9.4 | 9 |
| *Setonix brachyurus* | Quokka | VU | < 10000 | Australia | Northern Jarrah State Forest | 150 | 9.4 | 14 |
| *Setonix brachyurus* | Quokka | VU | < 10000 | Australia | Southern Jarrah State Forest | 700 | 9.4 | 66 |
| *Setonix brachyurus* | Quokka | VU | < 10000 | Australia | Bald Island Nature Reserve | 1000 | 9.4 | 94 |
| *Setonix brachyurus* | Quokka | VU | < 10000 | Australia | Rottnest Island Nature Reserve | 10000 | 9.4 | 945 |
| *Sminthopsis aitkeni* | Kangaroo Island Dunnart | CR | < 500 | Australia | Flinders Chase National Park | 425 | 9.4 | 40 |
| *Tapirus bairdii* | Baird's Tapir | EN | < 5500 | Colombia | Colombian protected areas | 250 | 7.6 | 19 |
| *Tapirus bairdii* | Baird's Tapir | EN | < 5500 | Nicaragua | Nicaraguan protected areas | 500 | 8.3 | 41 |
| *Tapirus bairdii* | Baird's Tapir | EN | < 5500 | Mexico | Mexican protected areas | 1500 | 5.9 | 89 |
| *Tapirus bairdii* | Baird's Tapir | EN | < 5500 | Honduras | Honduran protected areas | 500 | 25.0 | 125 |
| *Tapirus bairdii* | Baird's Tapir | EN | < 5500 | Panama | Panamanian protected areas | 1000 | 13.1 | 131 |
| *Tapirus bairdii* | Baird's Tapir | EN | < 5500 | Costa Rica | Costa Rican protected areas | 1000 | 18.2 | 182 |
| *Tapirus bairdii* | Baird's Tapir | EN | < 5500 | Guatemala | Guatemalan protected areas | 1000 | 30.8 | 308 |
| *Tapirus indicus* | Asian Tapir | EN | < 5000 | Thailand | Thailand, Western Forest Complex | 100 | 24.6 | 25 |
| *Urocyon littoralis* | Island Fox | CR | < 1500 | United States | Channel Islands National Park, San Miguel | 28 | 7.4 | 2 |
| *Urocyon littoralis* | Island Fox | CR | < 1500 | United States | Channel Islands National Park, Santa Rosa | 45 | 7.4 | 3 |
| *Urocyon littoralis* | Island Fox | CR | < 1500 | United States | Channel Islands National Park, Santa Cruz | 70 | 7.4 | 5 |
| *Ursus maritmus* | Polar Bear | VU | 20000-25000 | United States | USA protected areas (listed below) | 2000 | 7.4 | 147 |
| *Ursus maritmus* | Polar Bear | VU | 20000-25000 | United States | Bering Land Bridge National Preseve |  | 7.4 |  |
| *Ursus maritmus* | Polar Bear | VU | 20000-25000 | United States | Cape Krusenstern National Monument |  | 7.4 |  |
| *Ursus maritmus* | Polar Bear | VU | 20000-25000 | United States | Noatak National Preserve |  | 7.4 |  |
| *Ursus maritmus* | Polar Bear | VU | 20000-25000 | United States | Noatak Wilderness |  | 7.4 |  |
| *Ursus maritmus* | Polar Bear | VU | 20000-25000 | United States | Kobuk National Park |  | 13.7 |  |
| *Ursus maritmus* | Polar Bear | VU | 20000-25001 | Canada | Canadian protected areas (listed below) | 12904 | 13.7 | 1770 |
| *Ursus maritmus* | Polar Bear | VU | 20000-25000 | Canada | Ivvavik National Park |  | 13.7 |  |
| *Ursus maritmus* | Polar Bear | VU | 20000-25000 | Canada | Vuntut National Park |  | 13.7 |  |
| *Ursus maritmus* | Polar Bear | VU | 20000-25000 | Canada | Banks Island Bird Sanctuary 1 |  | 13.7 |  |
| *Ursus maritmus* | Polar Bear | VU | 20000-25000 | Canada | Aulavik National Park |  | 13.7 |  |
| *Ursus maritmus* | Polar Bear | VU | 20000-25000 | Canada | Tuktut Nogait National Park |  | 13.7 |  |
| *Ursus maritmus* | Polar Bear | VU | 20000-25000 | Canada | Quttinirpaaq National Park |  | 13.7 |  |
| *Ursus maritmus* | Polar Bear | VU | 20000-25000 | Canada | Polar Bear Pass National Wildlife Area |  | 13.7 |  |
| *Ursus maritmus* | Polar Bear | VU | 20000-25000 | Canada | Queen Maud Gulf Bird Sanctuary |  | 13.7 |  |
| *Ursus maritmus* | Polar Bear | VU | 20000-25000 | Canada | Katannilik Territorial Park |  | 13.7 |  |
| *Ursus maritmus* | Polar Bear | VU | 20000-25000 | Canada | Dewey Soper Bird Sanctuary |  | 13.7 |  |
| *Ursus maritmus* | Polar Bear | VU | 20000-25000 | Canada | Ukkusiksalik National Park |  | 13.7 |  |
| *Ursus maritmus* | Polar Bear | VU | 20000-25000 | Canada | Kaskatamagan Wildlife Management Area |  | 13.7 |  |
| *Ursus maritmus* | Polar Bear | VU | 20000-25000 | Canada | Wapusk National Park |  | 13.7 |  |
| *Ursus maritmus* | Polar Bear | VU | 20000-25000 | Canada | Polar Bear Provincial Park |  | 13.7 |  |
| *Ursus maritmus* | Polar Bear | VU | 20000-25000 | Canada | Iqualirtuuq National Wildlife Area |  | 13.7 |  |
| *Ursus maritmus* | Polar Bear | VU | 20000-25000 | Canada | Sirmlik National Park |  | 13.7 |  |
| *Ursus maritmus* | Polar Bear | VU | 20000-25000 | Canada | Bylot Island Bird Sanctuary |  | 13.7 |  |
| *Ursus maritmus* | Polar Bear | VU | 20000-25000 | Canada | Auyuittuq National Park |  | 7.4 |  |
| *Varecia variegata* | Black-and-white Ruffed Lemur | CR | < 10000 | Madagascar | Manombo Special Reserve | 133 | 5.0 | 7 |
| *Varecia variegata* | Black-and-white Ruffed Lemur | CR | < 10000 | Madagascar | Nosy Mangabe Reserve | 224 | 5.0 | 11 |
| *Varecia variegata* | Black-and-white Ruffed Lemur | CR | < 10000 | Madagascar | Mananara-Nord National Park | 1610 | 5.0 | 80 |
| *Varecia variegata* | Black-and-white Ruffed Lemur | CR | < 10000 | Madagascar | Ranomafana National Park | 2912 | 5.0 | 145 |
| *Varecia variegata* | Black-and-white Ruffed Lemur | CR | < 10000 | Madagascar | Zahamena National Park | 2961 | 5.0 | 148 |
|  |  |  |  |  |  |  |  |  |
