# Supplementary material for: A Population Accounting Approach to Assess Tourism Contributions to Conservation of IUCN-Redlisted Mammal Species
Source: PLoS One. 2012 Sep 12;7(9):e44134. doi: 10.1371/journal.pone.0044134 (PMC3440393; doi:10.1371/journal.pone.0044134)
Supplement: Table S2 — Proportions of tourism revenue in protected area budgets, by country. (DOC) [file pone.0044134.s002.doc]

Supplementary Table 2: The proportion of tourism revenue to protected area budgets as applied to protected areas and threatened species subpopulations by country in the current analysis.

Bovarnick, A., Fernandez-Baca, J., Galindo, J. & Negret, H. *Financial Sustainability of Protected Areas in Latin America and the Caribbean: Investment Policy Guidance*. United Nations Development Programme (UNDP) and The Nature Conservancy (TNC). New York. (2010)

Guha, I., & Ghosh, S. A glimpse of the tiger: how much are Indians willing to pay for it? South Asian Network for Development and Environmental Economics (SANDEE) Working Paper No. 39-09. Kathmandu. (2009)

Mansourian, S., & Dudley, N. *Public Funds to Protected Areas.* WWF International. (2008)

Turpie, J., Barnes, J., Longcamp, M. & Paxton, M. *Sustainable Financing Plan for Namibia’s Protected Area System: February 2010*. Ministry of Environment and Tourism, Directorate of Parks and Wildlife Management. Windhoek. (2010)
